# Gracillin Shows Potential Efficacy Against Non-Small Cell Lung Cancer Through Inhibiting the mTOR Pathway

**DOI:** 10.3389/fonc.2022.851300

**Published:** 2022-03-22

**Authors:** Yamei Li, Hai Liu, Xiaoxuan Liu, Bang Xiao, Minhong Zhang, Yaoling Luo, Mingchun Li, Jianqiong Yang

**Affiliations:** ^1^ The Clinical Medicine Research Center of the First Clinical Medical College, Gannan Medical University, Ganzhou, China; ^2^ College of Pharmacy, Gannan Medical University, Ganzhou, China; ^3^ National Engineering Research Center for Modernization of Traditional Chinese Medicine-Hakka Medical Resources Branch, Gannan Medical University, Ganzhou, China; ^4^ School of Rehabilitation Medicine, Gannan Medical University, Ganzhou, China; ^5^ Department of Oncology of the First Clinical Medical College, Gannan Medical University, Ganzhou, China

**Keywords:** gracillin, non-small cell lung cancer, autophagy, mTOR signaling pathway, anti-tumor

## Abstract

The leading cause of cancer deaths is lung cancer, non-small cell lung cancer (NSCLC), the most common type of lung cancers, remains a difficult cancer to treat and cure. It is urgent to develop new products to treat NSCLS. Gracillin, extracted from *Reineckia carnea*, *Dioscorea villosa*, and other medicinal plants, has anti-tumor potential with toxic effect on a variety of tumor cells such as NSCLC. However, the anti-NSCLC mechanism of gracillin is not completely clear. In this study, A549 cells and athymic nude mice were used as models to evaluate the anti-NSCLC effects of gracillin. The antiproliferative activity of gracillin on A549 cells was conducted by CCK-8, and obvious autophagy was observed in gracillin-treated A549 through transmission electron microscopy. Furthermore, the expressions of Beclin-1, LC3-II, and WIPI1 were upregulated, while the expression of p62 was downregulated in gracillin-treated A549. The further mechanism study found that the mTOR signaling pathway was significantly inhibited by gracillin. Accordingly, the PI3K/Akt pathway positively regulating mTOR was inhibited, and AMPK negatively regulating mTOR was activated. Meanwhile, LC3-II transformation was found to be significantly reduced after WIPI1 was silenced in A549 cells but increased after gracillin treatment. It also proves that WIPI is involved in the process of gracillin regulating A549 autophagy. At last, the anti-tumor growth activity of gracillin *in vivo* was validated in A549-bearing athymic nude mice. In conclusion, gracillin has anti-NSCLC activity by inducing autophagy. The mechanism maybe that gracillin inhibited the mTOR signaling pathway. Gracillin has the potential to be a candidate product for the treatment of NSCLC in the future.

## Introduction

Lung cancer is still one of the most common malignant tumors so far and the leading cause of cancers-related deaths in the world (1–3). According to data released in 2018, 1.8 million patients worldwide have died from lung cancer and the number of lung cancer patients increases sharply each year (4). NSCLC accounts for about 85% of lung cancers, and more than half of patients with lung cancer are diagnosed as NSCLC at the advanced stage of the disease (5). The current treatment methods for NSCLC mainly include surgery, radiotherapy, chemotherapy, immunotherapy, targeted therapy, etc. (6).However, the cumulative survival rate of patients with NSCLC within 5 years is still very low, only 16.8% (4). With the advent of precision medicine, great breakthroughs have been made in immunotherapy and targeted therapy for NSCLC, such as programmed death receptor 1/programmed death receptor ligand 1 inhibitors (PD-1/PD-L1 inhibitors) (7) and epidermal growth factor-tyrosine kinase inhibitors (EGFR-TKI) (8). Although these have contributed greatly to the treatment of lung cancer, they are not applicable to all patients. In addition, drug resistance and adverse reactions exist in the clinical treatment process, so that it is difficult to achieve the expected curative effect (9, 10). Therefore, it is necessary to find safe and effective new drugs for the treatment of NSCLC.

Traditional Chinese medicine (TCM) has a long history, and it has been reported that many compounds in TCM have strong antitumor activity, such as paclitaxel (11), triptolide (12), etc. It may be an efficient and fast way to find effective drugs against NSCLC from TCM. Gracillin is a steroidal saponin compound and is found in a variety of plants including *Rhizoma paridis* (13), *Pairs polyphylla* (14)*, Dioscorea villosa* (15)*, Acontum carmichaeli* (16), *Solanum incanum*, and *Solanum xanthocarpum* (17). According to reports, gracillin has anti-tumor, anti-inflammatory (18), pro-apoptotic (19), anti-bacterial (20) and other pharmacological effects. In particular, its potent antitumor effects *in vivo* and *in vitro* have attracted widespread attention. Chen found that gracillin induces human leukemia HL60 cell apoptosis and cell cycle G1 block *via* oxidative stress pathway (21); Min found that gracillin disrupts complex II-mediated mitochondrial function by inactivating succinate dehydrogenase, which resulted in decreased mitochondrial membrane potential, oxidative phosphorylation, and ATP production, at the same time, increased mitochondrial ROS production (22). And then they found that gracillin exerts a powerful anti-tumor effect mainly by inhibiting the production of bioenergy mediated by glycolysis and oxidative phosphorylation in tumor cells (23).Yang found that Gracillin exhibits a powerful anti-colorectal cancer effect by inhibiting the STAT3 pathway (24). The anti-tumor potential of gracillin is obvious, but there are relatively few studies on anti-tumor mechanisms, mainly involving apoptosis and energy metabolism. Our previous study found that gracillin can induce apoptosis of A549 cells by up-regulating Bax, casepase3, cytochrome C and down-regulating Bcl-2, which is related to the regulation of mitochondrial pathway (25). The Bcl-2 protein family plays a key role in regulating apoptosis and autophagy. It has been demonstrated that the autophagy-related protein Beclin-1 with BH3 domain can directly regulate autophagy by binding to Bcl-2 (26). In this context, we performed high-throughput screening of gracillin-treated A549 cells and found that many differentially expressed genes were enriched on autophagy-related regulatory networks. Therefore, we wondered whether gracillin affects A549 cell proliferation by regulating autophagy.

Autophagy, known as type 2 programmed cell death (27), functions to degrade damaged and redundant organelles and misfolded proteins, providing the molecular building and energy source to keep cells alive (28, 29). However, excessive autophagy can lead to autophagic cell death (30). mTOR is a very important regulator in the regulation of autophagy. mTOR inhibition has significant activity against a broad range of human cancers *in vitro* and in human tumor xenograft models (31). In this study, based on high-throughput screening, we found that WD repeat domain phosphoinositide-interacting protein 1 (WIPI1) was significantly highly expressed. And WIPI1 was reported as an autophagy-related gene (32). The prerequisite for the formation of autophagosomes is phosphatidyl alcohol-3-phosphate (PtdIn3p), which requires the participation of WIPI protein to function on the autophagosome membrane. WIPI represent the human proteome in the PROPPIN protein family, which fold into seven-bladed β-propeller proteins that bind PtdIn3P (33). Therefore, we first determined the antiproliferative effect of gracillin on A549 cells, and then confirmed that it could induce autophagy in A549 cells. Then, we studied the molecular mechanism of gracillin-activated autophagy and explored the role of WIPI1. Finally, the antitumor effect of gracillin was determined in athymic mice bearing A549 cells. Gracillin may be one of the drug candidates for the treatment of non-small cell lung cancer in the future.

## Materials and Methods

### Antibodies and Regents

**Figure 1:**
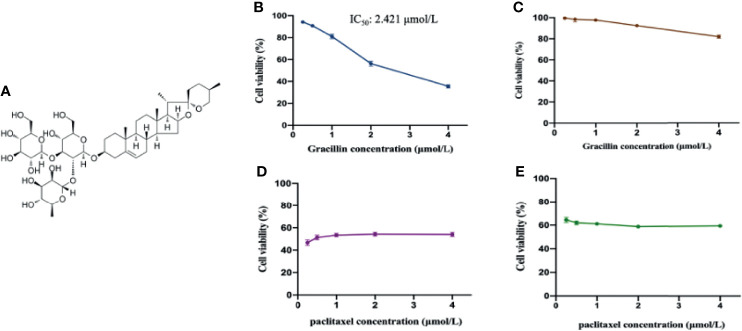
The anti-proliferative effect of gracillin on A549 cells and BEAS-2B cells. **(A)** The chemical structure of gracillin (PubChem CID: 159861). **(B)** The cytotoxicity of gracillin on A549 cells treated for 24 hours. **(C)** The cytotoxicity of gracillin on BEAS-2B cells for 24 hours. **(D)** The cytotoxicity of paclitaxel on A549 cells for 24 hours. **(E)** The cytotoxicity of paclitaxel on BEAS-2B cells for 24 hours.

Gracillin (HPLC > 98%) was isolated from *Reineckia carnea* and confirmed to be a steroidal saponin compound through structural identification, with the molecular formula C_45_H_72_O_7_ (**Figure 1A**). The compound was dissolved in dimethyl sulfoxide (DMSO) for experiments. The antibodies against SQSTM/P62, Beclin-1, LC3B, Atg12, PI3K kinase, phospho-PI3K kinase, Akt, phospho-Akt, AMPK and phospho-AMPKα were purchased from Cell Signaling Technology. The antibody against GAPDH was purchased from Beijing Apply Gene Technology Co., Ltd. The antibody against WIPI1 was purchased from Abcam Co. The horseradish peroxidase (HRP)-conjugated goat anti-mouse IgG, HRP-conjugated goat anti-rabbit IgG, the mTOR antibody, phospho-mTOR antibody, and the ultra-wide molecular weight marker (10 - 310 KDa) were purchased from Proteintech. The protein marker (10 - 180 KDa), Matrigel and Lipofectamine 2000 Reagent were purchased from Thermo Fisher Scientific Co. The WIPI1 forward primer (5’-TGCCATCACCTTCAATGCCTCAG-3’) and the reverse primer (5’-GCCATCCAGCGAAACCCAGAC-3’) were synthesized from Shanghai Sangon Biological Engineering Co., Ltd. The Trizol lysate was purchased from Ambion Co. The q-PCR kit and cDNA reverse transcription kit were purchased from Beijing Transgen Biotechnology Co., Ltd. The RIPA strong lysate, blasticidin and rapamycin were purchased from Beijing Solarbio Technology Co., Ltd. The PEGFP-LC3B plasmid was purchased from Wuhan Miaoling Biotechnology Co., Ltd. The cell counting kit-8 (CCK-8) was purchased from Glpbio Co. Human WIPI1 small interfering RNA (siRNA) lentivirus was constructed from Shanghai Novo Biotechnology. 3-Methyladenine(3-MA) was purchased from Med Chem Express Co.

### Cell Culture

A549 cells and BEAS-2B cells were purchased from the cell bank of the Type Culture Collection Committee of the Chinese Academy of Sciences (Shanghai, China). The cells were cultured in F12K medium supplemented with 10% FBS, 100 U/mL penicillin and 100 µg/mL streptomycin. All cells were grown in a humidified atmosphere at 37°C and 5% CO_2_.

### Cell Proliferation Assay

Exponentially growing A549 cells were obtained and seeded in a 96-well plate with 3×10^3^ cells per well. When the cell growth density reaches more than 70%, four groups were set up, namely the control group, the experimental group, the positive control group (paclitaxel), and the blank group. The cells of the experimental group are treated by gracillin (0.25, 0.5, 1, 2, 4 μmol/L). After 24 hours, the old medium was discarded, and 100 μL of fresh F12K complete medium and 10 μL of CCK-8 reaction solution were added. Then, the 96-well plate was placed in a 37°C constant temperature incubator and incubated for 2 hours. Finally, varioskan™ flash multimode reader (Thermo Fisher Scientific, Waltham, MA, USA) was used to determine the absorbance at 450nm. In addition, gracillin was further evaluated for its cytotoxic effect on human normal lung bronchial epithelial cells (BEAS-2B). The IC_50_ value of gracillin for 24 hours is calculated by the Logit method. The cell survival rate is calculated as follows:


$$\begin{array}{c} \text{cell survival rate (}\%)=(\text{the average OD value of the experimental group}–\text{the average OD value of the blank group})\\ /(\text{the average OD value of the control group}–\text{the average OD value of the blank group}) \end{array}$$

### Experiment of Inhibiting or Promoting Autophagy on the Proliferation of A549 Cells

First, the logarithmic growth A549 cells were seeded in a 96-well plate with 3×10^3^ cells per well. Secondly, when the cell confluence reaches more than 70%, the cells were treated by gracillin together with the 3-MA or Rapamycin. 3-MA is an inhibitor of phosphatidylinositol 3-kinase, and specifically blocks autophagosome formation in Autophagy. Rapamycin is an inhibitor of mTOR, which can switch the autophagy-specific protein phosphorylase mode to promote autophagy. The CCK-8 method was used to detect cell proliferation. All experiments were repeated at least 3 times.

### Morphological Observation Experiment of Autophagosome

Detecting autophagy-related structures in the cytoplasm by transmission electron microscopy (TEM) is the gold standard for determining autophagy. 2% glutaraldehyde fixation was used to fix the sample overnight, then the sample was post-fixed, filmed, TEM filming and image collection in the electron microscope room. The changes in organelles such as Golgi complex, endoplasmic reticulum, lysosome, and mitochondria in the cytoplasm were observed to determine whether autophagosomes with independent double-layer membrane structures were formed.

### pEGFP-LC3 Plasmid Transfection

Exponentially growing A549 cells were obtained and seeded in a laser confocal dish with 1×10^5^ cells When the cell confluence reached about 70%, pEGFP-LC3 plasmid was transfected into A549 cells using Lipofectamine 2000 (Invitrogen, Carlsbad, CA, USA) following the manufacturer’s instructions. After 6 - 8 hours of transfection, it was replaced with a new medium and placed in a 37°C, 5% CO2, saturated humidity incubator for 24 hours. The transfected cells were treated by autophagy inhibitor (3-MA) and gracillin for 24 hours, and then stained with DAPI for 10 minutes. The fluorescent spots were observed under a Zeiss carlzeis lsm880 confocal laser microscope.

### Western Blot Analysis

Cells and tumor tissues are lysed on ice for 30 minutes with RIPA lysis buffer containing protease inhibitors (PMSF) and phosphatase inhibitors. Equal amounts of the total protein (20 μg for cells, 50 μg for tissues) were separated by 8% - 15% SDS-PAGE gel. After electrophoresis, the proteins on the SDS-PAGE gel were transferred to the polyvinylidene fluoride membrane (PVDF). Then, the membrane was blocked for 1 hour in a TBST solution containing 5% skimmed milk powder or Bovine Serum Albumin (BSA) at room temperature. Furthermore, the membrane was incubated overnight with the different primary antibodies at 4°C. Next, the membrane will be further incubated for 1 hour with horseradish peroxidase (HRP) conjugated secondary antibody at room temperature. Finally, the protein bands on the membrane were visualized by Super Enhanced chemiluminescence detection reagents (Applygen Technologies Inc., Beijing, China) and detected by Chemi Dox XRS chemiluminescence imaging system (Bio-Rad, California, USA). The protein bands were quantified by Image J (NIH, USA) and the relative expression of the target protein was calculated by using GAPDH as an internal control. All data comes from three independent experiments.

### Quantitative Real-time PCR (qRT-PCR)

qRT-PCR is usually used to quantify the expression changes of gene at the mRNA level. A549 cells were seeded in 6-well plate with 2×10^5^ cells per well and placed in 37°C, 5% CO_2_ incubator. When growth density is greater than 70%, the cells were treated by gracillin (0.25, 0.5, 1, 2 μmol/L) for 24 hours. Trizol was used to extract the total RNA of the cells according to the instructions, and the concentration was measured by ultra-micro-UV spectrophotometer (Thermo Scientific, Massachusetts, USA). Then, the total RNA was reverse transcribed into cDNA with Easy Script ^®^ one-step gDNA removal and cDNA synthesis supermix kit (TransGen Biotech, Beijing, Chain). Subsequently, the cDNA was amplified and monitored in real time with the Perfect StartTM Green qPCR SuperMix kit (TransGen Biotech, Beijing, Chain) and CFX ConnectTM fluorescent quantitative PCR detection system (Bio-Rad, California, USA). The PCR primers of WIPI 1 are shown in “2.1”. The thermal cycling conditions are 94°C for 30 seconds; 94°C for 5 seconds; 60°C for 15 seconds; 72°C for 10 seconds; 40 cycles. The relative quantification of genes was analyzed according to the 2-ΔΔCt method [ΔΔCt = ΔCt (treatment)-ΔCt (control)]. GAPDH was used as an endogenous control. Each sample was analyzed three times.

### Transfecting A549 Cells With WIPI1 Knockout Lentivirus

The shRNA sequence targeting the human WIPI1 gene was inserted into the PDS126 vector to generate the WIPI1-si plasmid. The sequence that silences WIPI1 expression is 5’-GCTCTCTAGTGTTCAGTATGG-3’. First, logarithmic growth A549 cells were seeded in a 6-well plate with 2×10^5^ cells/well. When cell confluence reached 80%, the lentiviral particles targeting WIPI1 were transfected into cells with the optimal multiplicity of infection (MOI=10) for transfection. 6 - 8 hours after transfection, the transfection efficiency was observed by fluorescence microscope. Then, the blasticidin medium containing 16μg/mL was cultured continuously for 3 weeks, and the medium was changed every 3 days. Finally, a stably transfected cell line was screened out. The total RNA and total protein of the stable cell line were extracted, and the interference efficiency of the lentivirus was detected by q-PCR and Western blot. Then, the medium containing 4 μg/mL blasticidin was used to maintain a stable environment, and the selected cells were re-seeded in a 6-well plate at 2×10^5^ cells/well. Simultaneously one control group (empty vector-containing lentivirus-infected group) and five experimental groups with different concentrations gracillin (0, 0.25, 0.5, 1, 2 μmol/L) were set up. When the cell growth density reached 70% or more, the cells were treated by gracillin for 24 hours. Finally, the total cell protein was collected.

### Animal Experiment

The animal experiments were conducted under the guidance of the Animal Protection and Ethics Committee of Gannan Medical University. Forty BALB/c mice (4-6 weeks, 18-20 g), male and female, were purchased from Hunan Slack Jingda Experimental Animal Co., Ltd. (SCXK (Xiang) 2019-0004), 4 mice per cage. Mice are raised under standard animal feeding conditions (12 hours light/dark cycle) and controlled ambient temperature (25 ± 2°C) with free access to standard mouse food and water. All mice were acclimatized to the above-mentioned environment for one week before the start of the experiment. For the cell line xenotransplantation experiment, each mouse was inoculated with 5×10^6^ A549 cells in the right armpit of the mouse. Starting from the observable tumor tissue, the tumor volume was measured with a vernier caliper every day. the tumor volume was calculated by using the following formula: tumor volume (mm^3^) = (short diameter)^2^ × (long diameter) × 0.5. When the tumor volume reached 100mm^3^, the optimized mice were randomly divided into 5 groups (n=8), including a negative control group, three gracillin treatment groups and a positive control group. The three gracillin treatment groups were treated by different concentrations of gracillin (high dose 20 mg/Kg, medium dose 10 mg/Kg, low dose 5 mg/Kg) for 6 days a week for two consecutive weeks. In the negative control group, the mice were treated by solvent (corn oil and 5% DMSO) in the same way. The positive control group was treated by paclitaxel, three times a week for two weeks. During the treatment, the body weight and tumor volume of the mice were measured daily. Last, all mice were sacrificed, tumor tissues were collected, weighed, and photographed. Also, the tumor tissue was frozen in liquid nitrogen or immediately fixed in formalin for further study.

### Hematoxylin and Eosin (H&E) Staining

The heart, liver, kidney, and lungs of the mice were fixed with 4% paraformaldehyde and embedded in paraffin and sectioned. The slides were first deparaffinized and hydrated, then stained with hematoxylin solution and eosin solution, respectively, followed by dehydration, and finally, mounted. Representative images were obtained by the digital slice scanning analysis system Tissue FAXS plus (Tissue Gnostics, Austria).

### Ki67 Immunohistochemical Staining

Harvested tumors were paraffin-embedded and analyzed by immunohistochemistry. Tumor cell proliferation was analyzed using an antibody against Ki-67. That is, the slides were first deparaffinized and hydrated, then subjected to antigen retrieval, followed by blocked with BSA blocking solution, incubated with an anti-mouse polyclonal antibody against Ki67 at 4°C, the next day, sequentially incubated with secondary antibodies and performed DAB staining. Finally, the slides were permanently mounted with neutral resin and the images were recorded by the digital slice scanning analysis system Tissue FAXS plus.

### Statistical Analysis

All data were expressed as mean ± SD. The data was analyzed by GraphPad Prism 8.0 (San Diego, California, USA). The difference between the two groups was analyzed using independent sample *t* test, and the difference between multiple groups was analyzed using one-way analysis of variance (ANOVA) test. All the data provided have been verified by at least three independent experiments. When *P* ≤ 0.05, the difference was considered to have statistically significant.

## Results

### Gracillin Inhibits the Proliferation of A549 Cells

In this study, CCK-8 was used to study the anti-proliferative effects of gracillin in A549 cells and BEAS-2B cells. After treatment of gracillin for 24 hours, the results showed that gracillin significantly inhibited the cell viability of A549 in concentration-dependent manner (**Figure 1B**) with half maximal inhibitory concentration (IC_50_) value of 2.421 μmol/L. Meanwhile, gracillin had almost no effect on the proliferation of BEAS-2B cells (**Figure 1C**). Although paclitaxel showed a strong antiproliferative effect on the proliferation of A549 cells (**Figure 1D**), it also showed the same effect on the proliferation of BEAS-2B cells (**Figure 1E**). In general, gracillin had strong proliferation inhibitory effect on human NSCLC cells and had little effect on the proliferation of normal lung epithelial cells.

### Gracillin-Induced Autophagic Cell Death in A549 Cells

**Figure 2:**
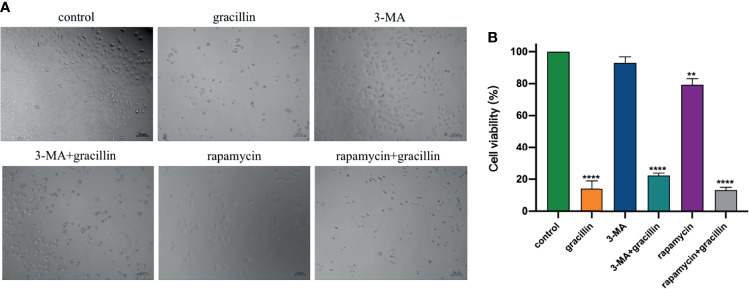
Anti-proliferation effect of gracillin in combination with 3-MA or rapamycin on A549 cells. **(A)** The growth of cells in 6 different treatment groups observed under the microscope. **(B)** The statistical results of the inhibition rate of the 6 groups on A549 cells, compared with the control group, **P ≤ 0.01, ****P ≤ 0.0001.

In this study, CCK-8 was used to examine the effect of gracillin-induced autophagic death in the presence of autophagy inhibitors and agonists. The results show that the number of dead cells in the rapamycin and gracillin together treatment group was greater than the gracillin (2 μmol/L) treatment group. At the same time, the number of dead cells in the gracillin treatment group was greater than the 3-MA and gracillin together treatment group. The number of dead cells in the 3-MA or rapamycin treatment group was relatively small, but the number of dead cells in the former was smaller than that in the latter (**Figure 2**). In summary, gracillin can induce autophagy in A549 cells and cause cells death.

### Observation of Autophagosomes by Transmission Electron Microscope and Laser Scanning Confocal Microscope

**Figure 3:**
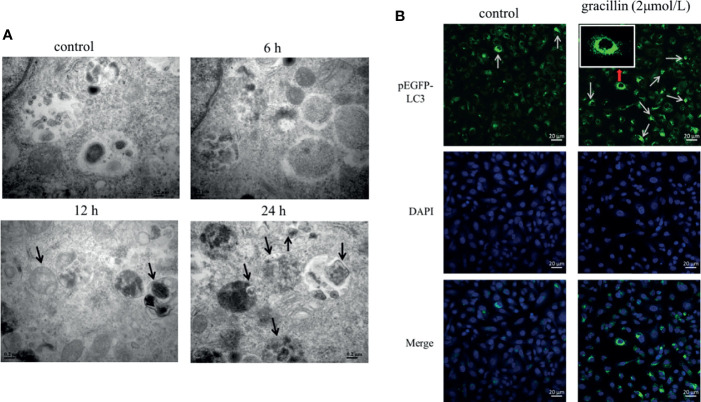
The effect of gracillin on autophagosome production. **(A)** Transmission electron microscope image of A549 cells treated with gracillin. The arrow indicates the autophagosome or autolysosome. **(B)** Confocal laser image of A549 cells transfected with pEGFP-LC3 plasmid and treated with gracillin. The nucleus was stained with DAPI. The arrow in the figure indicates the punctate aggregation of LC3 on the autophagosome membrane.

A549 cells were treated by 2μmol/L gracillin for 6 h, 12 h, and 24 h, respectively. The autophagosomes were observed by transmission electron microscopy. It was found that the autophagosomes were observed in the gracillin-treated group compared with the control group (**Figure 3A**). Observing the LC3 punctate aggregation is also one of the indicators to judge the occurrence of autophagy. LC3 protein is widely present in cells, when autophagy occurs, LC3 protein aggregates on the surface of autophagosomes and transforms into a punctate distribution. Because pEGFP-LC3 carries green fluorescent label, it can be judged whether the cells have autophagy by observing the degree of punctate aggregation of green fluorescent by Laser Scanning Confocal Microscope. After the pEGFP-LC3 plasmid was transfected into the cells, the LC3 punctate aggregation of the gracillin-treated cells could be observed, while the control group cells rarely had LC3 punctate aggregation (**Figure 3B**).

### Gracillin Affects the Expression of Autophagy-Related Proteins

**Figure 4:**
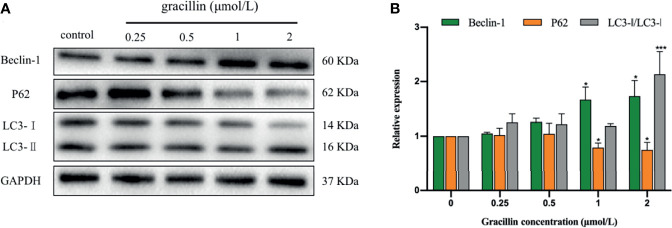
The effect of gracillin on autophagy-related proteins in A549 cells. **(A)** Western blot analysis of Beclin-1, P62, LC3-Iand LC3-II and A549 cells treated with gracillin (0, 0.25, 0.5, 1, 2 μmol/L) for 24 hours. The full picture is in **Supplementary Figure 1**. **(B)** The relative protein expression levels of Beclin-1, P62, LC3-I, and LC3-II were quantified by normalization to GAPDH, compared with the control group, *P ≤ 0.05, ***P ≤ 0.001.

After treated by gracillin for 24 hours, the expression changes of Beclin-1, P62 and LC3 in A549 cells were detected by Western blot. The results showed that the expression of Beclin-1 was significantly up-regulated, the expression of P62 was significantly down-regulated. The transformation from LC3-I to LC3-II was significantly increased. In general, gracillin could change the expression of autophagy-related proteins in A549 cells (**Figure 4**).

### The Expression of WIPI1 at Gene and Protein Levels

**Figure 5:**
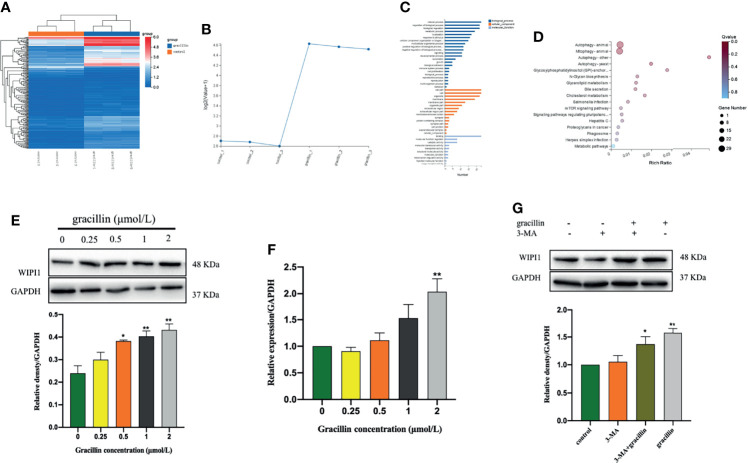
Gracillin caused changes in the expression of WIPI1 in A549 cells. **(A)** Cluster analysis of all differential gene expression in A549 cells treated with gracillin 2 μmol/L for 24 hours. **(B)** Differentially high expression of WIPI1. **(C)** GO function enrichment analysis, WIPI1 is enriched to the biological functions involved in the formation of autophagosome membranes. **(D)** KEGG Pathway enrichment analysis, WIPI1 is enriched into the signal pathway related to animal autophagy. **(E, F)** The differential expression of WIPI1 was verified by q-PCR and Western blotting. **(G)** The effect of combined use of gracillin and 3-MA on the expression of WIPI1. The full picture is in **Supplementary Figure 2**. Compared with the control group, *P ≤ 0.05, **P ≤ 0.01.

The bioinformatics analysis of gracillin-treated A549 cells showed that the gene WIPI1 is significantly differently expressed (**Figure 5A**) and can bind to phosphatidyl alcohol-3-phosphate, which is necessary for the formation of autophagosomes, through differential expression of genes Cluster analysis (**Figure 5B**), GO function enrichment analysis (**Figure 5C**) and KEGG pathway enrichment analysis (**Figure 5D**). Then we verified the results of bioinformatics analysis by q-PCR (**Figure 5E**) and Western blot (**Figure 5F**), and the results showed that the gracillin-treated A549 cells were up-regulated at both protein and gene levels. When 3-MA inhibited autophagy, the expression of WIPI1 was down-regulated, but the cells treated by 3-MA and gracillin (2μmol/L) together, the expression of WIPI1 was up-regulated (**Figure 5G**).

### Gracillin Reverses the Autophagy Inhibitory Effect of 3-MA

**Figure 6:**
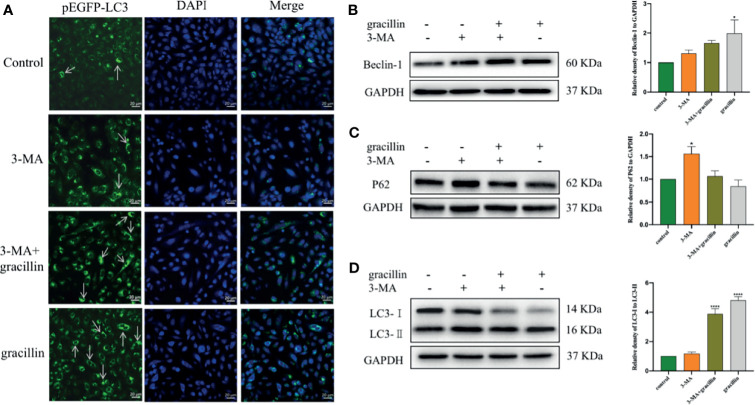
After 3-MA inhibits autophagy, the effect of gracillin on the degree of autophagy in A549 cells and the protein expression of Beclin-1, P62, LC3-Iand LC3-. **(A)** Confocal laser image of gracillin-treated A549 cells. The nucleus was stained with DAPI. The arrow in the figure indicates the punctate aggregation of LC3 on the autophagosome membrane. One of the fluorescent punctation represents an autophagosome. **(B)** Western blot analysis of Beclin-1 and relative protein expression levels were quantified by normalization to GAPDH. **(C)** Western blot analysis of P62 and relative protein expression levels were quantified by normalization to GAPDH. **(D)** Western blot analysis of LC3 and relative protein expression levels are expressed as LC3-II/LC3-I. The full picture is in **Supplementary Figure 3**. Compared with the control group, *P ≤ 0.05, ****P ≤ 0.0001.

3-MA blocks the formation of autophagosomes by inhibiting the class III phosphatidylinositol 3-kinases (PI3K). Under the Laser Scanning Confocal Microscope, it can be observed that the degree of punctate aggregation with green fluorescent (pEGFP-LC3) was significantly reduced in A549 cells of 3-MA treatment group. But it was significantly increased in cells treated with 3-MA and gracillin together (**Figure 6A**). The extent to which autophagy is inhibited by 3-MA can be reversed by gracillin, which is achieved by up-regulating Beclin-1(**Figure 6B**), promoting the transformation of LC3 to LC3-II (**Figure 6D**), and down-regulating P62 (**Figure 6C**).

### Gracillin Inhibits mTOR Signaling Pathway

**Figure 7:**
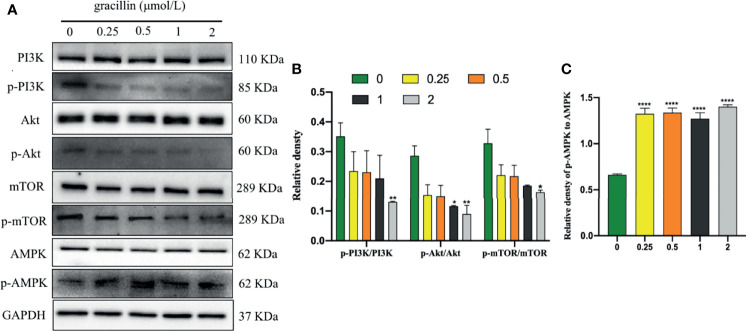
Gracillin induces autophagy in A549 cells through the mTOR signaling pathway. **(A)** Western blot analysis of PI3K, p-PI3K, Akt, p-Akt, AMPK, p-AMPK, mTOR, p-mTOR and GAPDH in A549 cells treated with gracillin (0, 0.25, 0.5, 1, 2 μmol/L) for 24 hours. **(B)** The relative protein expression levels of p-PI3K, p-Akt, and p-mTOR are quantified using PI3K, Akt, and mTOR as standards. **(C)** The relative protein expression level of p-AMPK is quantified with AMPK as standard. The full picture is in **Supplementary Figure 4**. Compared with the control group, *P ≤ 0.05, **P ≤ 0.01, ****P ≤ 0.0001.

The kinase mTOR plays a key role in the progress of autophagy and activates autophagy by inhibiting phospho-mTOR (p-mTOR). mTOR is a downstream target for PI3K/AKT, and AMPK, the activation of which suppresses autophagy. Inhibit phospho-PI3K (p-PI3K) and phospho-Akt (p-Akt) or activate phospho-AMPK (p-AMPK) can cause p-mTOR to be inhibited to activate autophagy. In this study, after A549 cells were treated with gracillin for 24 hours, the total protein levels of PI3K, Akt, AMPK, and mTOR were not significantly changed. p-PI3K, p-Akt and p-mTOR were significantly down-regulated, and p-AMPK was significantly up-regulated (**Figure 7**). In general, gracillin could activate autophagy by inhibiting mTOR signaling pathway.

### WIPI1 Participates in Gracillin-Induced Autophagy

**Figure 8:**
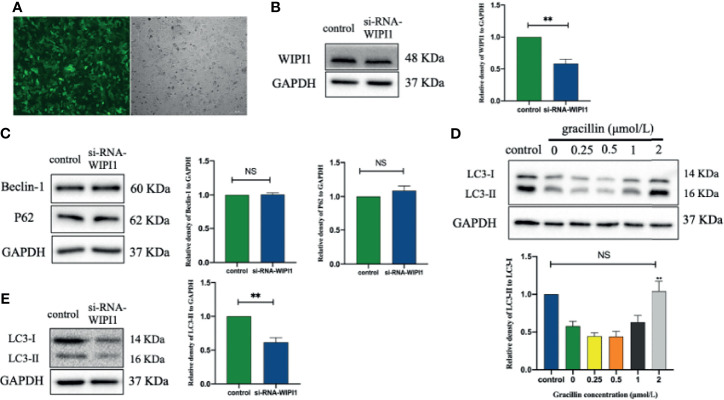
The effect of gracillin on autophagy in A549 cells with WIPI1 gene silenced. **(A)** Fluorescence microscope image of A549 cells with WIPI1 silenced (MOI = 10). **(B)** Western blot verified the effect of WIPI1 being silenced and the relative protein expression level of WIPI1 was quantified by normalization to GAPDH. **(C)** Western blot and relative protein expression levels of Beclin-1 and P62 were quantified by normalization to GAPDH. **(E)** Western blot and relative protein expression level of LC3 are expressed as LC3-II/LC3-I. **(D)** The effect of gracillin on LC3-Iand LC3-II in A549 cells with WIPI1 gene silenced. The full picture is in **Supplementary Figure 5**. Compared with the control group, **P ≤ 0.01, NS *P* > 0.05.

After transfecting A549 with a lentivirus targeting WIPI1, the cells can be observed with green fluorescence under a fluorescence microscope, and the stable cells can be screened by blasticidin (**Figure 8A**). At the same time, the expression of WIPI1 in stable cells was detected and found that the expression of si-RNA-WIPI1 group was significantly interfered compared with the control group (**Figure 8B**). In addition, we found that the protein expression of Beclin-1 and P62 were not affected by WIPI1 (**Figure 8C**), while the protein expression of LC3 was significantly affected (**Figure 8E**). To verify whether WIPI1 is involved in gracillin-induced autophagy, we treated the stable cells with gracillin, and then collected proteins for Western blot experiment. The results showed that although gracillin showed an up-regulation trend for LC3-II, the up-regulation trend was not obvious compared with the control group (**Figure 8D**). In other words, silencing WIPI1 influences gracillin-induced autophagy.

### Gracillin Inhibits NSCLC Tumor Growth in Xenograft Model

**Figure 9:**
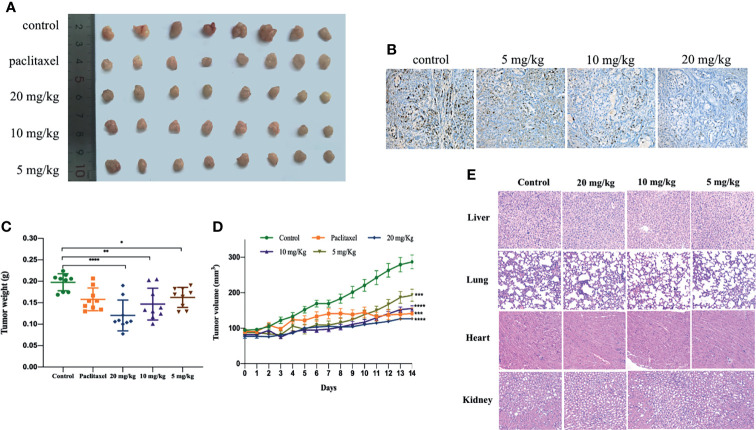
Gracillin inhibits tumor growth in nude mouse models induced by A549. After tumor formation, mice were treated by intraperitoneal injection of gracillin (5, 10, 20 mg/kg) for 2 weeks (n = 8, female and male). **(A)** The mice tumor picture; **(B)** Ki67 immunohistochemical staining; **(C)** tumor volume; **(D)** tumor weight; **(E)** H&E stained image of liver, lung, heart and kidney of gracillin-treated mice. *P ≤ 0.05, **P ≤ 0.01, ***P ≤ 0.001, ****P ≤ 0.0001.

We further verified the anti-tumor effect of gracillin by establishing tumor xenograft model. When the tumor growth reached approximately 100mm^3^, the mice were treated with gracillin (20 mg/kg, 10 mg/kg, or 5 mg/kg) six days a week for two consecutive weeks. The results clearly showed that the tumor volume growth rate of gracillin-treated mice was significantly lower than that of the control group with dose-dependence (**Figures 9A, D**). At the same time, the tumor weight of the mice in the gracillin-treated groups were also significantly reduced (**Figure 9C**). Meanwhile, Ki67 immunohistochemical staining showed that the proliferation of tumor cells was significantly inhibited by gracillin (**Figure 9B**).

In addition, we also extracted the liver, lung, heart, and kidney of the mice and performed H&E staining. The results showed that gracillin had no obvious toxicity to the liver, lung, heart, and kidney of the mice (**Figure 9E**). The specific manifestations are as follows: hepatic cords are arranged regularly, and cells are clearly stained; alveoli are evenly distributed and structured, with a small amount of connective tissue between adjacent alveoli; myocardial tissue is stained uniformly and clearly, cells are arranged neatly and densely, and muscle fibers are aligned and arranged regularly; the structure of the glomerulus is clear, and the shape of the renal tubules is regular.

In conclusion, gracillin has been shown to have an effective inhibitory effect on tumor growth in athymic nude mice carrying A549. In summary, gracillin has shown potential inhibitory effect on tumor of A549 bearing athymic nude mice.

## Discussion

According to the latest research report, lung cancer has the highest mortality rate among all cancers (34). As the most classic subtype of lung cancer, NSCLC has malignant metastasis potential and low cure rate (35). Curing NSCLC remains a huge challenge so far. Therefore, it is necessary to find effective and safety drugs. In this experiment, A549 cells, which have been extensively used in the study of NSCLC were employed, were selected as study subjects. Now many compounds derived from TCM displayed potent anti-tumor effect in many cancers. Based on our previous study, we found that a steroidal saponin, gracillin, has significant anti-proliferation activity in A549 cells. Gracillin has been validated to inhibit the proliferation of various tumor cells (19, 21). However, there are relatively few studies on its anti-tumor mechanism. In this study, gracillin was used as a drug candidate against NSCLC, and its anti-tumor effect and mechanism were explored through cell models and animal models.

In this study, the proliferation of A549 cells was significantly inhibited by gracillin, but the proliferation of BEAS-2B cells was inhibited to a lesser extent. In addition, when A549 cells were treated by 3-MA or rapamycin together with gracillin, it was found that the anti-proliferative effect of gracillin in combination with rapamycin was stronger than that in combination with 3-MA, indicating that gracillin Positive induction of autophagy in A549 cells. Autophagy plays a vital role in cancer and has become a potential target for cancer treatment (36). In this study, we found that there are obvious double-layer membranes or multilayer membrane structures in gracillin-treated A549 cells, which indicates that gracillin activates autophagy. The same result can be obtained by observing the pEGFP-LC3 punctate aggregation. At the same time, gracillin induces autophagy by up-regulating Beclin-1, promoting LC3-II transitions, and down-regulating P62. We also found that when 3-MA inhibited autophagy in A549 cells, it could be reversed gracillin-treated A549 cells. Beclin-1 is the mammalian homolog of yeast Atg6 and plays a key role in controlling Vps34-mediated vesicle transport and autophagy induction (37). P62 is an important selective autophagy adaptor protein, which participates in the process of removing ubiquitinated protein as a receptor and transport ubiquitinated protein to the proteasome for degradation (38). LC3 is a marker of the autophagy process. After the synthesis of LC3 protein, the C-terminal 5 peptides are cleaved by Atg4, the glycine residues are exposed, and cytoplasmic localized LC3-I is produced. When autophagy occurs, LC3-I will be modified and processed by ubiquitin-like systems including Atg7 and Atg3 and coupled with phosphatidylethanolamine (PE) to form LC3-II and localize on the inner and outer membranes of autophagy (39). Therefore, the degree of autophagy could be judged by observing the pEGFP-LC3 punctate aggregation on the autophagosome membrane by laser confocal microscope.

Based on the results of high-throughput screening, Further research found that WIPI1 was significantly overexpressed at the mRNA level in gracillin-treated A549 cells. WIPI1 is a new gene that has the potential to become an autophagy marker. It is a homolog of Atg18, located in autophagosomes and early endosomes, and plays an important role in the migration of macrophages (40, 41). The formation of autophagosomes in mammals is related to endosomes, and WIPI1 specifically acts in the formation and fission of tubulo-vesicular endosomal transport carriers (42). In addition, when 3-MA inhibited autophagy, the expression of WIPI1 protein was down-regulated, but this phenomenon could be reversed by gracillin, indicating that WIPI1 is closely related to autophagy in A549 cells. Because WIPI1 has a typical seven-bladed β-propeller structure that can bind to PI3P produced downstream of mTOR (33), the difference in the expression of WIPI1 caused by gracillin may be related to mTOR-related signaling pathways. In this research, it was proved that gracillin inhibits mTOR signaling pathway through down-regulate the expression of p-PI3K and p-Akt and up-regulate the expression of p-AMPK. In addition, found that silencing WIPI1 would reduce the expression of LC3-II, thereby reducing autophagy. But this phenomenon can be alleviated by gracillin, which may be caused by the inability of WIPI1 to be completely silenced. But this phenomenon can be alleviated by gracillin, which may be because WIPI1 cannot be completely silenced.

To study the anti-tumor effect of gracillin in NSCLC, we established a tumor model of athymic nude mice carrying A549. It was found that the tumor growth rate of mice with gracillin treatment was slower than the control group, and the high dose of gracillin (20 mg/kg) has a better anti-tumor effect compared with the paclitaxel treatment group. At the same time, we found that the paclitaxel-treated mice died or became emaciated in the late treatment period, whereas the gracillin-treated mice did not. From the H&E staining results of liver, lung, heart, and kidney of mice, it can be found that gracillin is basically non-toxic to mice, and literature reports can also prove this result (24). Study on the anti-tumor mechanism, absorption, and metabolism of gracillin *in vivo* will become the direction of our future research.

## Conclusion

**Figure 10:**
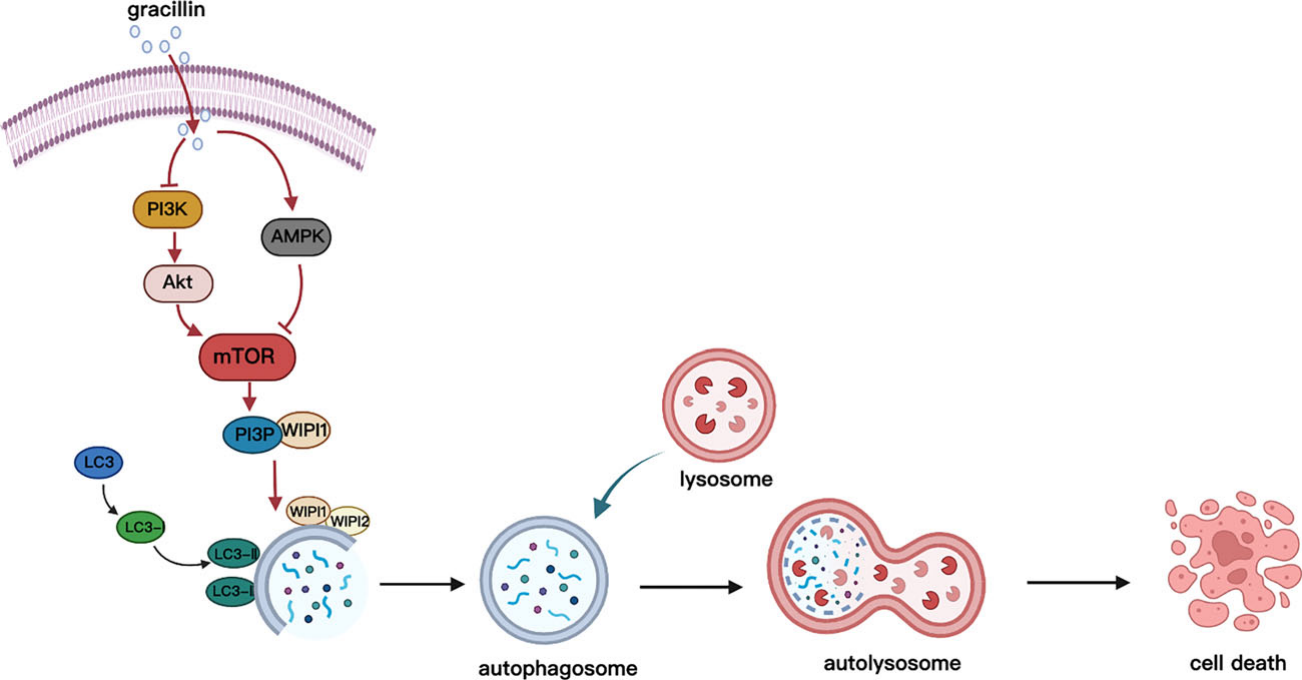
Schematic diagram of gracillin inducing autophagic death of A549 cells. Gracillin regulates the mTOR signaling pathway in A549 cells to induce autophagy death. Gracillin inhibits p-mTOR by inhibiting p-PI3K and p-Akt and activating p-AMPK. WIPI1 is involved in the downstream regulation of autophagy by mTOR.

In conclusion, this study found that gracillin has anti-tumor effects on NSCLC, and its anti-tumor mechanism is the regulation of autophagy through the mTOR signaling pathways (**Figure 10**). We cautiously proposed that gracillin has great potential in the treatment of NSCLC.

## Data Availability Statement

The original contributions presented in the study are included in the article/**Supplementary Material**. Further inquiries can be directed to the corresponding author.

## Ethics Statement

The animal study was reviewed and approved by the Animal Protection and Ethics Committee of Gannan Medical University.

## Author Contributions

JY and HL conceived and designed the experiments, contributed new reagents and analysis tools, and supervised all the research. YLL, XL, and BX performed the experiments. YML wrote the original manuscript. YML, MZ, ML, and YLL analyzed the data. JY and HL revised the manuscript. All authors contributed to the article and approved the submitted version.

## Funding

This work was supported by the National Natural Science Foundation of China (No. 31460082), Key Projects of Traditional Chinese Medicine Science and Technology Plan of Jiangxi Province (2021Z016), Science and Technology Project of Jiangxi Provincial Health Commission (202210946), Key R&D Project of Ganzhou Science and Technology Plan (202101124809).

## Conflict of Interest

The authors declare that the research was conducted in the absence of any commercial or financial relationships that could be construed as a potential conflict of interest.

## Publisher’s Note

All claims expressed in this article are solely those of the authors and do not necessarily represent those of their affiliated organizations, or those of the publisher, the editors and the reviewers. Any product that may be evaluated in this article, or claim that may be made by its manufacturer, is not guaranteed or endorsed by the publisher.
